# Unraveling the impact of lanthanum on methane consuming microbial communities in rice field soils

**DOI:** 10.3389/fmicb.2024.1298154

**Published:** 2024-01-23

**Authors:** Ruyan Liu, Ziting Wei, Wanying Dong, Rui Wang, Jonathan M. Adams, Lin Yang, Sascha M. B. Krause

**Affiliations:** ^1^School of Ecology and Environmental Sciences, East China Normal University, Shanghai, China; ^2^School of Geographic and Oceanographic Sciences, Nanjing University, Nanjing, China

**Keywords:** methane, soil health, rare earth elements, ecotoxicology, microbial communities, lanthanum, global change

## Abstract

The discovery of the lanthanide requiring enzymes in microbes was a significant scientific discovery that opened a whole new avenue of biotechnological research of this important group of metals. However, the ecological impact of lanthanides on microbial communities utilizing methane (CH_4_) remains largely unexplored. In this study, a laboratory microcosm model experiment was performed using rice field soils with different pH origins (5.76, 7.2, and 8.36) and different concentrations of La^3+^ in the form of lanthanum chloride (LaCl_3_). Results clearly showed that CH_4_ consumption was inhibited by the addition of La^3+^ but that the response depended on the soil origin and pH. 16S rRNA gene sequencing revealed the genus *Methylobacter, Methylosarcina*, and *Methylocystis* as key players in CH_4_ consumption under La^3+^ addition. We suggest that the soil microbiome involved in CH_4_ consumption can generally tolerate addition of high concentrations of La^3+^, and adjustments in community composition ensured ecosystem functionality over time. As La^3+^ concentrations increase, the way that the soil microbiome reacts may not only differ within the same environment but also vary when comparing different environments, underscoring the need for further research into this subject.

## 1 Introduction

Methane (CH_4_) is an important greenhouse gas after carbon dioxide (CO_2_) with a global warming potential of 85 or 32 for 20- or 100-y time horizons, respectively (IPCC, 2021). Rice fields stand out as one of the major anthropogenic contributors to atmospheric CH_4_, accounting for 14.1 to 48% of total agricultural CH_4_ emissions (Carlson et al., 2017; Saunois et al., 2020; Gupta et al., 2021; Li et al., 2022; Duan et al., 2023; Qian et al., 2023).

Methylotrophs constitute a microbial group that can utilize single-carbon or multi-carbon compounds without carbon–carbon bonds, including methanol and methylamine, for growth and as energy source (Vu et al., 2021). CH_4_-oxidizing bacteria (methanotrophs) are a special case in this group because they are additionally capable of utilizing CH_4_ as their primary carbon and energy source (Hanson and Hanson, 1996). In rice fields, methanotrophs can oxidize up to 90% of the CH₄ before it reaches the atmosphere (Conrad and Rothfuss, 1991). Consequently, methanotrophs play a vital role as a biofilter, significantly contributing to the regulation of the global climate in agro-ecosystems like rice fields.

Lanthanides, a group of 15 elements from lanthanum (La^3+^, *Z* = 57) to lutetium (*Z* = 71) on the periodic table, along with scandium and yttrium, collectively known as Rare Earth Elements (REEs), have garnered increased interest over the past four decades. Their pivotal role in modern technology, serving as essential components in portable electronic devices and clean energy technologies, has fueled this growing interest (Leal Filho et al., 2023). Notably, REEs are also of interest in agriculture, being used as additives in animal feed to enhance livestock yield and as components in fertilizers to boost crop productivity (Balaram, 2019; Tommasi et al., 2021). REEs can be introduced to the soil through phosphate fertilizers, contributing to their accumulation in the soil (Silva et al., 2019; Dushyantha et al., 2020). For instance, the concentration of La^3+^ in the soil can vary widely from 1.1 to 413 mg/kg, but also can reach exceptionally high levels in areas close to mining areas, ranging from 1800.44 to 6905.24 mg/kg (Tyler, 2004; Li et al., 2010; Liang et al., 2014; Ramos et al., 2016).

Lanthanides were long considered biologically inactive due to their low bioavailability (Bulman, 2003) but a biological significance has recently been discovered. In the first step of CH_4_-oxidation, CH_4_ is converted to methanol which is then further oxidized to formaldehyde by an enzyme called methanol dehydrogenase (MDH). Calcium (II) ions have been thought of as the cofactor of MDHs (Anthony, 2000), but a novel form of MDHs is requiring lanthanides (Hibi et al., 2011; Pol et al., 2014; Skovran and Martinez-Gomez, 2015; Picone and op den Camp, 2019; Kato et al., 2020). It was demonstrated that the newly discovered functional MDH is primarily expressed even in the presence of 95 μM Ca^2+^ in the cultivation medium and with as little as 1 μM supplemental La^3+^ (Chu and Lidstrom, 2016). Despite these discoveries in physiology, there is still a knowledge gap for the role and function of La^3+^ in natural and agroecosystems. It is largely unknown whether methanotrophic communities in soils are inhibited or stimulated by the addition of La^3+^.

Because the diversity of methanotrophs is limited, elevated levels of La^3+^ in the soil may lead to a loss of function that could not be compensated by other microbes with dramatic consequences for atmospheric CH_4_ concentrations and global warming. However, La^3+^ is an important element in the metabolism of methanotrophs, and increasing concentrations in the environment could be beneficial with a positive effect on ecosystem processes, bioremediation, and biotechnological applications (Skovran and Martinez-Gomez, 2015; Dance, 2023). To the best of our knowledge, only Kravchenko and colleagues (Kravchenko et al., 2022) have examined the implications of La^3+^ addition on the composition and activity of methanotrophs in sod-podzolic soil (pH = 5.4). The addition of 5 μg La^3+^ stimulated the growth of the methanotrophic genus *Methylobacter* and the non-methanotrophic genus *Methylotenera*. In addition, a stimulating effect on CH_4_ oxidation was observed at 200 parts per million (ppm) in the headspace of their experimental setup (Kravchenko et al., 2023).

In this study, we focused on the impact of increasing amounts of La^3+^ in rice field soil. Therefore, we performed a comprehensive lab incubation study combining gas chromatography, mass spectrometry, sequencing, and quantitative real-time polymerase chain reaction (qPCR) to get a better understanding of the ecological implications of the increasing amounts of REEs on the soil microbiome, which plays a crucial role in mitigating atmospheric CH_4_ emissions. We selected three rice field soils with different pH conditions to explore the relationship between the soil microbiome and the bioavailability and mobility of La^3+^ within this soil ecosystem. Previous studies clearly demonstrated the direct impact of pH on the solubility, mobility, and bioavailability of La in the soil (Iftekhar et al., 2018; Kotelnikova et al., 2021; Zhao et al., 2021). We hypothesized that La^3+^ in the form of lanthanum chloride (LaCl_3_) will stimulate methanotrophic activity and growth due to their availability to utilize lanthanides in their enzyme machinery. We further hypothesized that the soil origin and their specific soil properties (e.g., pH) will influence the La^3+^ retention capacity and consequently the effect on the soil microbiome.

## 2 Materials and methods

### 2.1 Study sites

In October 2020, three rice field soil samples with different pH (0–20 cm) were collected from Jiangsu Province, China, each originating from varying pH levels of 5.76, 7.2, and 8.36, representing acidic, neutral, and alkaline soils, respectively. Acidic soil samples were collected from Yangzhou City (32°24′ N, 119°2′ E). Neutral soil samples were collected from Xuzhou City (34°15′ N, 118°8′ E). Alkaline soil samples were collected from Yancheng City (33°17′ N, 120°34′ E). For incubation experiments, soil samples were stored at 4°C. However, soil physicochemical properties were determined from fresh or frozen soil (−20°C) except for the determination of La content in the original soil samples. All measured properties are summarized in Table 1. Detailed description of methods was published in a previous study (Guo et al., 2022) and carried out by Nanjing innovation biotechnology Co., LTD. The determination of La was carried out by TÜV Rheinland (Shanghai) Co., Ltd., China using Inductively Coupled Plasma Mass Spectrometry (ICP-MS).

**Table 1:** Main soil characteristics of the initial rice field soil samples.

Sample name soil properties	Acidic sample	Neutral sample	Alkaline sample
pH	5.67	7.2	8.36
La (mg/kg dry soil)	46.93	47.37	32.78
Conductivity (ms/cm)	0.09	0.1	0.35
Water content (fresh)	0.23	0.23	0.25
Organic matter (g/kg)	19.48	26.98	11.16
Organic carbon (g/kg)	11.3	15.65	6.47
Total phosphorus (P) (g/kg)	0.33	0.86	1.07
Total potassium (K) (g/kg)	10.71	13.77	18.65
Ca (mg/kg)	359.50	791.50	672.87
Cu (mg/kg)	2.33	3.10	1.45
Magnesium (Mg) (mg/kg)	2152.20	1167.82	273.91
N (g/kg)	1.34	1.76	0.79
Cl^−^ (g/kg)	0.05	0.08	0.32
NO_3_^−^ (mg/kg)	5.35	3.90	0.70
NH_4_^+^ (mg/kg)	2.17	2.25	3.86
Available P (mg/kg)	4.40	33.13	37.47

### 2.2 Experimental setup and sampling

In August 2022, 2 grams (*g*) of rice field soils and 25 milliliters (mL) of diluted nitrate mineral salt (NMS) Ca^2+^ medium (0.1 × strength, Dedysh and Dunfield, 2017) were mixed in 100 mL transparent serum bottles, these bottles were then placed in the shaker (MQT-60R, Shanghai Minquan Instrument Co., Ltd., China) and incubated in the dark at 20°C and 120 revolutions per minute (rpm) for 4 weeks. The diluted NMS medium consisted of 22.5 mL different concentrations of La^3+^ in the form of LaCl_3_ (Sangon Biotech (Shanghai) Co., Ltd., China) and 2.5 mL NMS (Ca^2+^) medium. To find suitable concentrations of La^3+^ for our experiment, we first searched the literature for ecotoxicological studies that applied La^3+^ (e.g., Chu et al., 2003; Li et al., 2018; Kotelnikova et al., 2019). We then chose LaCL_3_ concentrations ranging from 2 mg/L to 1,200 mg/L to determine the levels that depicted the strongest response in CH_4_ oxidation. Finally, the chosen concentrations included no added La^3+^ (control), 300 mg/L (low), 600 mg/L (intermediate), and 1,200 mg/L (high), denoted as control CK, L, M, and H levels, respectively.

All chemical reagents were purchased through Sangon Biotech (Shanghai) Co., Ltd., China. Each soil sample has four La^3+^ treatments in triplicate, 36 bottles in total. All bottles for the experiment were thoroughly washed with 1 mol/L hydrochloric acid (HCl) overnight and autoclaved before use, sealed with butyl rubber stoppers, and capped with plastic aluminum shells.

All 36 bottles were pre-incubated on a shaker at 20°C and 120 rpm in the dark for 24 h. During the experiment, on the first day of each week, the headspace of the bottles was flushed three times with air using a 60 mL syringe. Subsequently, 1.5 mL of 99.999% high-purity CH_4_ (Shanghai Jiaya Chemical Co., Ltd., China) was added resulting in a starting CH_4_ concentration of approximately 10,000 parts per million volume (ppmV) (~1%) in the headspace reflecting on typical *in-situ* methane concentrations in rice fields.

On the seventh day of each week, 2.5 mL of a well-mixed cultivation solution was extracted from the bottles. The extracted solution was then transferred to centrifuge at 7,800 rpm for 12 min using an Eppendorf Microcentrifuge 5,430 R (Eppendorf, Germany). Subsequently, the resulting supernatant underwent analysis for pH (S220-K, METTLER TOLEDO, Switzerland), and the remaining samples were stored in a − 80°C refrigerator for subsequent molecular analyses and La^3+^, NH_4_^+^, Ca^2+^, NO_3_^−^, and PO_4_^3−^ determination. The CH_4_ concentration in the headspace was determined using GC (Nexis GC-2030, Shimadzu, Japan) equipped with a Flame Ionization Detector (FID).

### 2.3 Determination of La^3+^ in the soil

The analysis of La^3+^ levels in the soil during the fourth week was carried out by Shanghai WEIPU Testing Technology Group Co., Ltd. (Shanghai, China). In brief, approximately 0.1 g of each soil sample was placed into a microwave digestion vessel and 4 mL of nitric acid was added for microwave digestion. Subsequently, each sample was tested using the Inductively Coupled Plasma-Optical Emission Spectrometer (ICP-OES) (PerkinElmer Avio 500, United States).

### 2.4 Determination of La^3+^, NH_4_^+^, Ca^2+^, NO_3_^−^, and PO_4_^3−^ ions in the supernatant

All analyses were performed by the Shanghai WEIPU Testing Technology Group Co., Ltd. (Shanghai, China). In brief, for the La^3+^ determination 0.1 mL of the supernatant was transferred into digestion tubes, followed by the addition of 4 mL of nitric acid. The tubes were then heated at 120°C for 4 h until the solution became clear and transparent. Subsequently, the digestive solution was adjusted to a constant volume of 10 mL. La^3+^ was determined using either ICP-MS (PerkinElmer NexION 2000, United States) or ICP-OES (PerkinElmer AvioVIO 500, United States), depending on the concentration range. NH_4_^+^, Ca^2+^, NO_3_^−^, and PO_4_^3−^ concentrations were determined by diluting each sample 10–20 times with water, and then the aqueous solution was filtered through a 0.22 μM membrane into a 2 mL Agilent vial and an injection tube for ion chromatography analysis using the Dionex ICS-5000^+^ ion chromatography system (Thermo Fisher Scientific, USA).

### 2.5 Soil DNA extraction and 16S rRNA gene analysis

The DNeasy PowerSoil^®^ Pro Kit (Qiagen, Germany) was used to extract total soil community genomic DNA from 0.2–0.25 g of each sample, following the manufacturer’s steps. The concentration of the DNA solution was checked through NanoDrop™ One Microvolume UV–Vis Spectrophotometer (Thermo Fisher Scientific, United States). The variable regions V1-V3 of bacterial 16S rRNA gene were amplified using the primers of 27F (AGRGTTTGATCMTGGCTCAG) (Lane, 1991) and 519R (GWATTACCGCGGCKGCTG) (Lane et al., 1985). The process of PCR amplification of the 16S rRNA gene, followed by library preparation, quantification, and sequencing was executed using the Illumina MiSeq platform (Illumina MiSeq, United States) at Sangon Biotech (Shanghai) Co., Ltd., China. The reaction system of PCR was used as follows: microbial DNA (10–20 ng/μL) 2 μL; amplicon PCR forward primer (10 μM) 1 μL; amplicon PCR reverse primer (10 μM) 1 μL; 2 × Hieff^®^ Robust PCR Master Mix (Yeasen, 10105ES03, China) 15 μL; 30 μL in total. The following PCR program was used: 1 cycle of denaturing at 94°C for 3 min, first 5 cycles of denaturing at 94°C for 30 s, annealing at 45°C for 20 s, elongation at 65°C for 30°C for 30 s, then 20 cycles of denaturing at 94°C for 20 s, annealing at 55°C for 20 s, elongation at 72°C for 30 s and a final extension at 72°C for 5 min.

16S rRNA gene high-throughput raw sequencing data was analyzed with the package of DADA2 version 1.26.0 (Callahan et al., 2016) in R 4.2.2 (Team, R. C, 2022). In brief, 16S rRNA gene forward and reverse reads were trimmed at positions 290 and 250 from the end or any position. Error models were constructed with random sample picking. The resulting amplicon sequence variances (ASVs) were taxonomically classified by using the SILVA ribosomal RNA database SSU version 138 (Pruesse et al., 2012). ASVs that were identified as mitochondrial or chloroplast sequences, or not classified into prokaryotic phyla were removed from the dataset. Removing ASVs less than 0.001% left 5,794 ASVs in the final dataset, 95.12% of total reads were retained.

### 2.6 qPCR of the *pmoA* gene

This experiment was conducted by Sangon Biotech (Shanghai) Co., Ltd., China. In brief, the *pmoA* gene was quantified using a specific qPCR assay to enumerate the total abundance of methanotrophs (MTOT assay; Kolb et al., 2003). The *pmoA* gene encodes the β-subunit of the particulate methane monooxygenase (pMMO) which catalyzes the first step in CH_4_-oxidation. It has been widely used as a functional marker to identify methanotrophs from the environment (Dedysh and Knief, 2018). The primer sequences were A189 F (GGN GAC TGG GAC TTC TGG) and mb661R (CCG GMG CAA CGT CYT TAC C) (Holmes et al., 1995; Costello and Lidstrom, 1999). In addition, this primer set was shown to have a broad coverage of methanotroph diversity (Bourne et al., 2001). The qPCR protocols for the *pmoA* gene were executed as follows: initial pre-denaturation at 95°C for 4 min; followed by denaturation at 95°C for 15 s; annealing at 67°C for 20 s; and extension at 72°C for 30 s. The previous steps comprised 10 cycles, with the annealing temperature decreasing by 1°C after each cycle. Subsequently, denaturation was carried out at 95°C for 15 s, followed by annealing at 57°C for 20 s, and extension at 72°C for 30 s, repeated for a total of 35 cycles. Copy numbers of target genes were derived from plasmid DNA. The PCR efficiency was on average 92.8% with an R^2^ value of 0.9972. Amplicon specificity was determined from melt curve analysis and confirmed by 1.5% agarose gel electrophoresis.

### 2.7 Statistical analysis

The CH_4_ concentration in the headspace was calculated by using a CH_4_ standard gas (1,002 ppmV) measured on the GC before sample measurements. *pmoA* gene copy numbers were expressed as per gram dry weight.

The Shannon index was calculated using the R package vegan 2.6.4 (Oksanen et al., 2022), with prior sequence flattening. The variation in microbial community compositions with different La^3+^ treatments and time points were assessed through non-metric multidimensional scaling (NMDS). First, ASVs with relative abundance below 0.05% were removed, reducing sparsity from 94 to 62.73%. Next, Bayesian-Multiplicative replacement of count zeros (cmultRepl) in the R package zCompositions package version 1.4.0.1 (Palarea-Albaladejo and Martín-Fernández, 2015) was employed to impute zeros in compositional count data. Then, a centered log-ratio (CLR) transformation was applied using R package compositions version 2.0.4 (van den Boogaart et al., 2022). For assessing the dissimilarity among various treatments and time points, Permutational multivariate analysis of variance (PERMANOVA) was performed using the function of adonis2 in the R package vegan version 2.6.4. NMDS plots at a 95% confidence level were generated using the “stat_ellipse” function in the R package ggplot2 version 3.4.1 (Wickham, 2016). R package reshape2 version 1.4.4 (Wickham, 2007), dplyr version 1.1.0 (Wickham et al., 2023), and tidyverse version 2.0.0 (Wickham et al., 2019) were utilized to process the data to calculate relative abundance plots. The ddply function in the R package plyr version 1.8.8 (Wickham, 2011) was employed to calculate the mean value ± standard deviation of pH in the supernatant. The leveneTest function in the R package car version 3.1.2 (Fox and Weisberg, 2019) was used to conduct the homogeneity of variance test of data. Analysis of variance (ANOVA) was performed using the stat_compare_means function in the R package ggpubr version 0.6.0 (Kassambara, 2023). Following that, *post hoc* tests were performed using the Least Square Difference (LSD) test in R package agricolae version 1.3.5 (de Mendiburu, 2021).

## 3 Results

### 3.1 CH_4_ Consumption is affected by the addition of La^3+^

**Figure 1:**
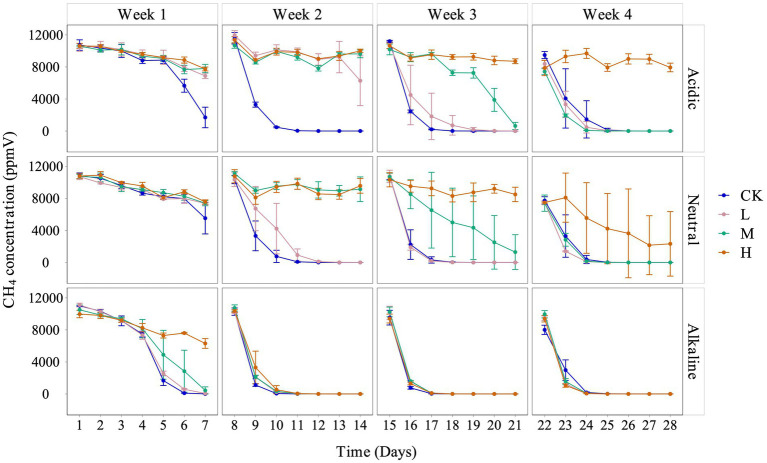
Variation of CH_4_ concentration in the headspace with different pH conditions for control (CK), 300 (L), 600 (M), and 1200 (H) mg/L La^3+^ treatments, from top to bottom are acidic, neutral, and alkaline samples, respectively. Error bars indicate the standard deviation (*n* = 3).

We observed a consistent trend of inhibited CH_4_ consumption across all treatments, with the degree of inhibition corresponding to the concentrations of La^3+^ added to the soil slurries. It is important to note that the extent of inhibition correlated with the pH of the soil slurries, each derived from rice fields with distinct acidity levels: acidic, neutral, and alkaline (Figure 1).

Although there was a clear inhibition of CH_4_ consumption, the data revealed an interesting trend: during the fourth week, in soil slurries from acidic and neutral rice fields, the low and intermediate treatments exhibited a faster rate of CH_4_ consumption between 22 and 24 days. This trend was also observed across all La treatments throughout the entire fourth week cultivation period, in soil slurries from alkaline rice fields (Figure 1).

### 3.2 The effect of La^3+^ on the methanotrophic community composition, microbial diversity, and methanotroph abundance

**Figure 2:**
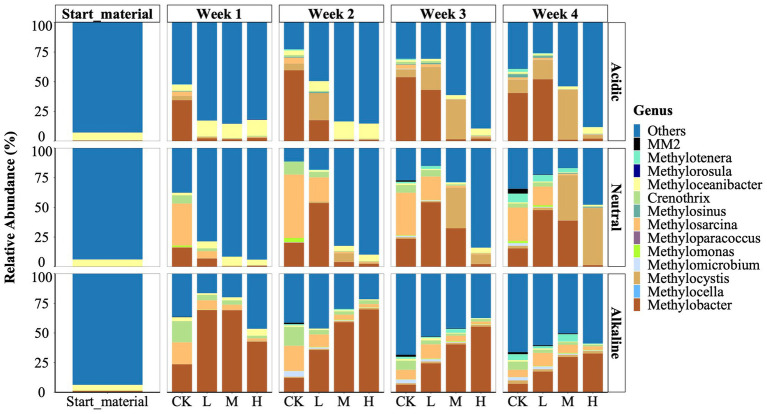
Relative abundance of the methylotrophs of starting material, control (CK), 300 (L), 600 (M), and 1200 (H) mg/L La^3+^ treatments in varying pH rice field soil samples, from top to bottom, respectively, are acidic, neutral, alkaline samples. Data is based on 16 s rRNA gene sequencing data (see Material and Methods for details).

We identified a total of 13 genera of methylotrophs using Illumina sequencing of the 16S rRNA gene (Figure 2). These types were distributed among five families: *Beijerinckiaceae* (comprising *Methylocella*, *Methylorosula*); *Crenotrichaceae* (*Crenothrix*); *Hyphomicrobiaceae* (*Methyloceanibacter*); *Methylococcaceae* (including *Methyloparacoccus. Methylobacter*, *Methylomicrobium*, *Methylomonas*, and *Methylosarcina*); *Methylocystaceae* (comprising *Methylocystis* and *Methylosinus*); and *Methylophilaceae* (*Methylotenera* and *Methylovorus* sp. strain MM2). Among these, *Methyloceanibacter*, *Methylorosula*, *Methylotenera*, and *Methylovorus* sp. strain MM2 were identified as facultative methylotrophs, while the remaining 9 genera were classified as obligate methanotrophs. Notably, *Methylocella*, *Methyloparacoccus*, and *Methylorosula* were absent from the neutral and alkaline soil samples.

We observed that the genus *Methyloceanibacter* was a prevalent microbe across the three initial rice field soil samples with a relative abundance of 6.3, 6.0, and 4.6% in the acidic, neutral, and alkaline samples, respectively (Figure 2). Representatives of *Methyloceanibacter* have been described to also oxidize CH_4_, but possess solely a SMMO, and were mainly found in marine environments (Vekeman et al., 2016).

We further observed that during the incubation period, the genera *Methylobacter*, *Methylocystis*, and *Methylosarcina* emerged as the dominant methanotrophs in the control treatment. However, their response to La^3+^ addition displayed variations among soil slurries with different initial pH levels: acidic, neutral, and alkaline (Figure 2). In both soil slurries from acidic and neutral rice fields, the application of higher doses of La^3+^ consistently resulted in lower relative abundances of the genus *Methylobacter*. For instance, in the treatments with high amount of added La^3+^ (referred to as the “H treatments”), *Methylobacter*’s relative abundance ranged from 0.9 to 2.6%. In contrast, in the corresponding control treatments (referred to as the “CK treatments”) for acidic samples, *Methylobacter*’s relative abundance remained notably higher, ranging from 34.4 to 59.6% (Figure 2). Once CH_4_ consumption was resumed (Figure 1) we also observed higher relative abundance of the genus *Methylobacter* (Figure 2). Intriguingly, when examining soil slurries from alkaline soil, we observed a distinct relationship between the relative abundance of the genus *Methylobacter* and the amount of added La^3+^. Specifically, in the CK and treatments with low, intermediate, and high levels of added La^3+^, the relative abundance of *Methylobacter* increased progressively between treatments, such as its abundance was 12.1% (CK), 35.7% (L), 59.1% (M), and 69.8% (H) in week 2 (Figure 2).

Next, we noticed a significant shift in the predominant methanotroph population from the genus *Methylobacter* (Type I) to the genus *Methylocystis* (Type II) in soil slurries from acidic and neutral rice field soils at higher La^3+^ concentrations (Figure 2). In soil slurries from the acidic rice field, *Methylobacter* dominated the CK treatments consistently throughout the cultivation process, while *Methylocystis* gradually took over in the M treatment after the second week. For instance, in the M treatments, *Methylocystis* showed a notably higher relative abundance during week 3 (33.4%) and week 4 (41.9%) compared to *Methylobacter*, which only accounted for 1.5% during week 3 and 1.2% during week 4. In soil slurries from the neutral rice field, *Methylocystis* progressively established as the dominant species within the M and H treatments. For instance, in week 4, *Methylocystis* showed a significantly higher relative abundance (48%) in the H treatment compared to *Methylobacter*, which had only 1.3% relative abundance.

In general, the relative abundance of non-methanotrophic taxa such as *Afipia*, *Pseudolabrys*, and *Pseudarthrobacter* increased with La^3+^ addition in acidic soil slurry (Supplementary Figure S1A). Noteworthy, in alkaline soil slurries, the *ASV6*-*Bdellovibrionaceae*-*Bdellovibrio* emerged as the most predominant non-methanotrophic organism. Notably, the *Bdellovibrio* genus is recognized as a bacterial predator (Feng et al., 2017). The relative abundance of the *ASV6*-*Bdellovibrionaceae*-*Bdellovibrio* in L treatment was higher than that in the CK treatment, Specifically, in the L treatments, its relative abundance was 12.9, 8.3, and 11.7% from week 2 to week 4, while in the CK treatments it was 7.2, 5.2, and 4.3%, respectively (Supplementary Figure S1C). Finally, in soil slurries that displayed inhibition of CH_4_ consumption (Figure 1) we observed that the community composition resembled its original inoculum suggesting that the community did not exhibit any growth (Supplementary Figure S1).

We used NMDS and PERMANOVA to provide further evidence for the observed shifts in community composition. NMDS plots clearly illustrated a significant effect of added La^3+^ on the composition of microbial communities in soil slurries from the acidic (*p* < 0.05), neutral (*p* < 0.001), and alkaline (*p* < 0.001) rice fields (Supplementary Figure S2).

The Shannon diversity index (SDI) was used to describe the response of the total microbial diversity to the addition of different amounts of La^3+^. The results showed that, in all experimental treatments including the control, the complexity of microbial communities simplified when compared to the starting material over the course of the experiment (Supplementary Figure S3). However, we observed that the diversity values for the H treatments of soil slurries from the acidic rice field in week 3 (SDI = 5.70) and week 4 (SDI = 5.52) nearly matched that of the initial material (SDI = 5.59). A similar trend was observed for the diversity values for the M (SDI = 5.37) and H (SDI = 5.48) in week 2 and the H (SDI = 5.38) treatment in week 3 of soil slurries from the neutral rice field. This similarity to the starting material indicated and artifact and suggested that the addition of La^3+^ likely inhibited the total microbial community (see Supplementary Figure S3).

**Figure 3:**
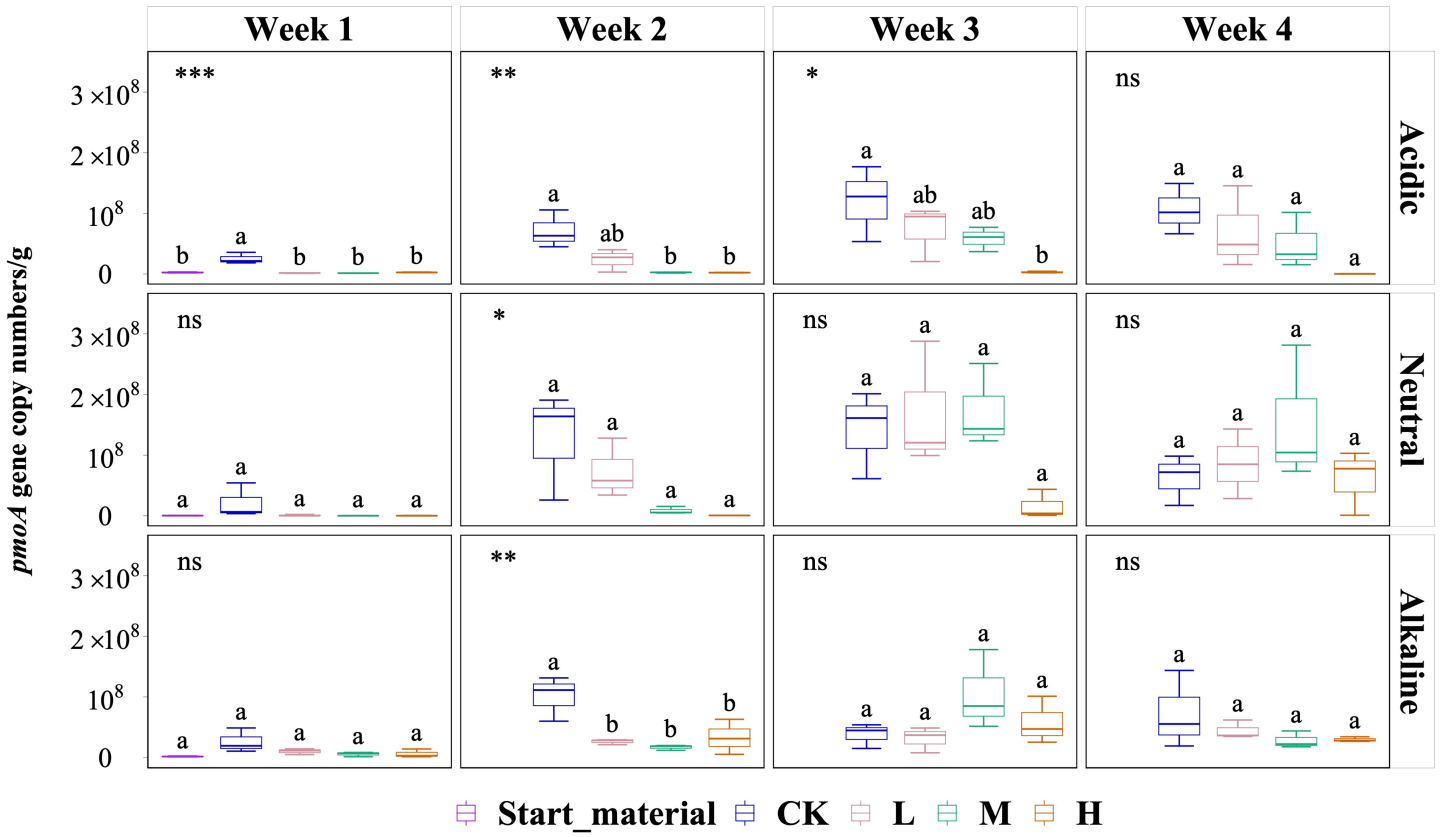
Variation of *pmoA* gene copy numbers (per g) in three samples with varied pH rice field soil conditions for start material, control (CK), 300 (L), 600 (M), and 1200 (H) mg/L La^3+^ treatments. Error bars indicate the standard deviation (*n* = 3) (*, *p* < 0.05; **, *p* < 0.01; ***, *p* < 0.001; ****, *p* < 0.0001; ns, no significance). Distinct letters in the plot indicate statistically significant differences among different La^3+^ treatments (*p* < 0.05).

Overall, *pmoA* gene copy numbers (per g) did not vary significantly by the end of the experiment (Figure 3). However, in soil slurries from the acidic rice field, a significant inhibitory effect of added La^3+^ was observed but this diminished in the last week of the experiment. For instance, during the second week, the *pmoA* gene copy numbers (per g) in the CK, L, M, and H groups were 7.12 × 10^7^, 2.35 × 10^7^, 2.38 × 10^6^, and 2.20 × 10^6^, respectively. In soil slurries from the neutral rice field, we observed no significant differences but observed a similar trend in the second week of the experiment. In the second week, *pmoA* gene copy numbers (per g) for the CK, L, M, and H groups were 1.27 × 10^8^, 7.33 × 10^7^, 8.49 × 10^6^, and 5.15 × 10^5^, respectively. In soil slurries from the alkaline rice field, we only observed a significant inhibition of added La^3+^ in the second week, the *pmoA* gene copy numbers (per g) in the CK, L, M, and H groups were 1.01 × 10^8^, 2.63 × 10^7^, 1.69 × 10^7^, and 3.32 × 10^7^, respectively (Figure 3).

### 3.3 Added La^3+^ affected pH in soil slurries and the retention of La^3+^ in soil particles

The pH in the supernatant indicated a clear relationship between added La^3+^ and acidity of the soil slurries from acidic and neutral rice fields (Table 2). In contrast, soil slurries from the alkaline rice field did not show any effect of added La^3+^ on the pH indicating a higher buffer capacity of that soil (Table 2).

**Table 2:** pH of the supernatant for control (CK), 300 (L), 600 (M), and 1200 (H) mg/L La^3+^ treatments in samples with different pH levels.

pH	Treatment	Week 1	Week 2	Week 3	Week 4
Acidic	CK	6.49 ± 0.27 a	7.06 ± 0.05 a	7.12 ± 0.05 a	6.97 ± 0.15 a
	L	5.16 ± 0.09 b	5.24 ± 0.39 b	4.57 ± 0.25 b	5.86 ± 0.23 b
	M	4.14 ± 0.17 c	4.4 ± 0.28 c	4.1 ± 0.14 c	4.76 ± 0.03 c
	H	4.1 ± 0.2 c	4.04 ± 0.24 c	3.95 ± 0.16 c	4.24 ± 0.18 d
Neutral	CK	7.25 ± 0.12 a	7.35 ± 0.07 a	7.63 ± 0.18 a	7.4 ± 0.17 a
	L	5.99 ± 0.99 ab	7.02 ± 0.03 a	7.03 ± 0.22 ab	7.3 ± 0.03 a
	M	5.59 ± 0.44 ab	5.31 ± 0.54 b	6.5 ± 0.5 b	6.8 ± 0.14 a
	H	4.54 ± 0.54 b	4.01 ± 0.05 c	4.2 ± 0.13 c	5.42 ± 0.52 b
Alkaline	CK	7.53 ± 0.14 b	7.63 ± 0.03 b	7.73 ± 0.23 a	7.74 ± 0.03 a
	L	7.85 ± 0.08 a	7.92 ± 0.02 a	7.78 ± 0.47 a	7.85 ± 0.03 a
	M	7.67 ± 0.13 ab	7.82 ± 0.06 a	7.8 ± 0.25 a	7.75 ± 0.02 a
	H	7.45 ± 0.07 b	7.7 ± 0.04 b	7.82 ± 0.12 a	7.54 ± 0.07 b

Mean ± SD is shown (*n* = 3), and distinct letters in the table indicate statistically differences among different La^3+^ treatments (*p* < 0.05).

**Figure 4:**
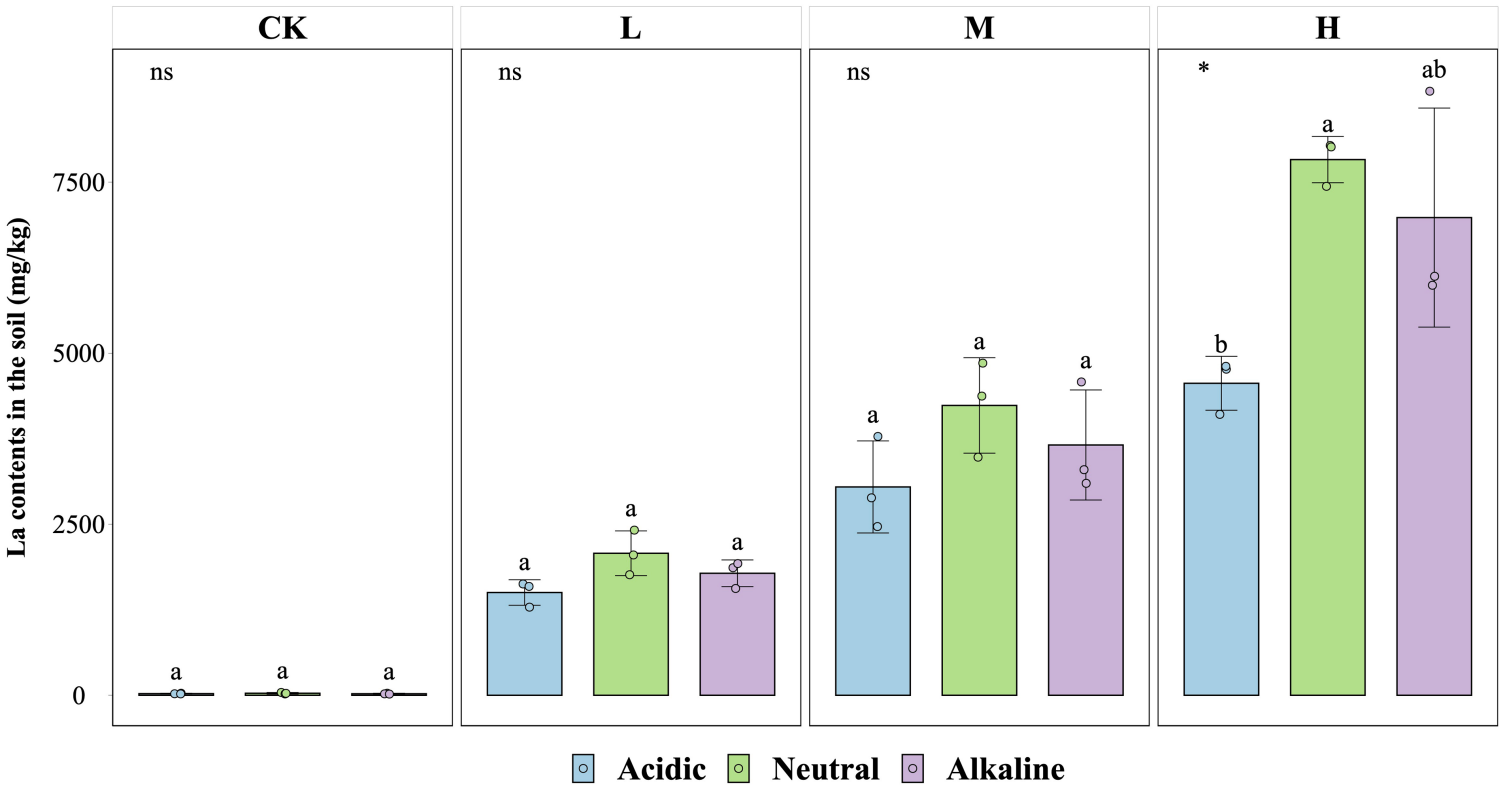
La contents in soil for the fourth week for control (CK), 300 (L), 600 (M), and 1200 (H) mg/L La^3+^ treatments (*, *p* < 0.05; **, *p* < 0.01; ***, *p* < 0.001; ****, *p* < 0.0001; ns, no significance). Error bars indicate SE (*n* = 3). Distinct letters in the plot indicate statistically differences among different pH samples (*p* < 0.05).

The concentration of La^3+^ in soil particles at the end of the experiment was similar across soil slurries from rice fields with different pH values (Figure 4). There were no significant differences in La^3+^ absorption capacity between CK, L, and M treatments from all soil types, except for soil slurries from the acidic rice field in the H treatment (*p* < 0.05). In this case the La^3+^ content reached values of 4,560 mg/kg (acidic soil condition), 7,830 mg/kg (neutral soil condition), and 6,982 mg/kg (alkaline soil condition).

We also measured the La^3+^ content in the supernatant at the end of each week of the experiment to identify patterns in the bioavailable La^3+^ (Supplementary Figure S4). No consistent patterns were observed. However, a notable discrepancy in La^3+^ concentration was observed between the H and the L treatments in soil slurries from acidic rice fields during weeks 2, 3, and 4. Specifically, the La^3+^ concentration in the H treatments measured 140.73 mg/L, 133.53 mg/L, 143.27 mg/L, respectively, surpassing that of the L treatments in week 2 (64.93 μg/L), week 3 (171.83 μg/L), and week 4 (191.33 μg/L).

In soil slurries from the neutral rice field, the La^3+^ concentrations in the CK and L treatments were below the detection limit of the machine (5 μg/L) but we observed a similar trend as seen before that the contents of La^3+^ in the H treatments far greater than the M treatments (Supplementary Figure S4). The La^3+^ concentrations in the H treatments were 16.77 mg/L (week 1), 14.98 mg/L (week 2), 13.67 mg/L (week 3), and 16.04 mg/L (week 4), whereas in the M treatments, the corresponding concentrations were 346 μg/L (week 1), 200.23 μg/L (week 2), 100.97 μg/L (week 3), and 326.87 μg/L (week 4). Moreover, when analyzing soil slurries from the alkaline rice field, it was evident that the La^3+^ content was lower compared to acidic and neutral samples. The concentrations of La^3+^ in week 1, in the L treatments in week 2 and 3, and in the M treatments in week 3, and CK treatments in week 4 were all below the detectable limit of the machine (5 μg/L). Interestingly, the La^3+^ concentration in solution displayed a positive correlation with the amount of added La^3+^ in week 4 (76.60, 170.83, and 228.93 μg/L for L, M, and H treatments, respectively).

### 3.4 NH_4_^+^, Ca^2+^, NO_3_^−^, and PO_4_^3−^ concentration changes in supernatant of soil slurries of different soil types

We also measured important soil chemical data in the first and last week of the experiment (Supplementary Figure S5). Firstly, concentration levels of NH_4_^+^ and PO_4_^3−^ in the supernatant were found to be below the detection limit of the machine (0.1 mg/kg and 0.5 mg/kg, respectively, Supplementary Figure S5). Secondly, NO_3_^−^ concentration levels did not show any notable differences in the first week. By week 4 an overall decrease was observed in all setups. In the supernatant of soil slurries from acidic rice fields the NO_3_^−^ concentration in week 4 decreased by 49.89% (CK), 44.58% (L), 31.85% (M), and 3.28% (H) compared to their respective concentrations in week 1. In the supernatant of soil slurries from neutral rice fields the NO_3_^−^ concentration decreased by 31.30, 50.96, 36.73, and 14.46% for CK, L, M, and H treatments, respectively. In the supernatant of soil slurries from alkaline rice fields the NO_3_^−^ concentration decreased by roughly 22.55, 45.63, 44.07, and 43.79% for CK, L, M, and H treatments, respectively. Finally, we observed a positive correlation between the Ca^2+^ concentration in the supernatant of soil slurries and La^3+^ dosage in week 1 and 4 of the experiment (Supplementary Figure S5). In week 4, Ca^2+^ concentration in the supernatant of soil slurries from the acidic rice field reached the following levels: 1.80 mg/kg (CK), 33.83 mg/kg (L), 72.29 mg/kg (M), and 103.77 mg/kg (H). In neutral rice field soil slurries, its contents were 28.60 mg/kg (CK), 49.74 mg/kg (L), 93.77 mg/kg (M), and 175.18 mg/kg (H). Ca^2+^ concentration in the supernatant of soil slurries from the alkaline samples reached the following levels in week 4: 40.48 mg/kg (CK), 56.40 mg/kg (L), 98.56 mg/kg (M) and 193.65 mg/kg (H) (Supplementary Figure S5).

## 4 Discussion

In this laboratory microcosm study, we aimed to gain a better understanding of the ecological implications of increasing amounts of REEs, in terms of the soil microbiome which plays a crucial role in mitigating CH_4_ emissions in agricultural soils. We specifically chose three rice field soils with different pH conditions because the pH is an important regulating factor for the bioavailability of La^3+^ in aquatic and soil ecosystems (Ran and Liu, 1993; Cao et al., 2001).

The addition of external La^3+^ changed the activity of CH_4_-consuming microbial communities in the soil slurries. We observed an inhibition of CH_4_ consumption based on the amount of added La^3+^, time, and soil origin. In line with the CH_4_ consumption data (Figure 1), the response of the soil microbiome followed a similar pattern with no significant changes in community composition from the starting material at higher concentrations of added La^3+^, especially in slurries with soil from acidic rice fields (Supplementary Figure S1). As such, our experimental data did not support our hypothesis that the soil microbiome, involved in CH_4_ consumption, will be stimulated by the addition of La^3+^ (Figure 2). Our data rather suggests that there are complex interactions between the soil origin, environmental conditions (e.g., pH), and the soil microbiome, involved in CH_4_ consumption. Hence, the soil microbiome’s response to increasing concentrations of La^3+^ may not only vary within a single ecosystem but also between ecosystems, calling for additional research on this topic.

One possible explanation for the inhibitory effect of La^3+^ on the soil microbiome in our experiment may be attributed to chemical interactions between this element and other significant elements important in microbial cellular functions. For instance, Ca^2+^ are versatile signaling molecules that impact almost every aspect of cellular life and play crucial roles in physiological functions such as cell migration and growth (Clapham, 2007; Giorgi et al., 2018). Numerous studies have reported analogous chemical properties between La^3+^ and Ca^2+^ owing to their closely matched ionic radius, charge, ligand specificities, and biological roles (e.g., Reeves and Condrescu, 2003). These similarities suggest that La^3+^ can effectively substitute Ca^2+^, with its trivalent ion displaying a higher affinity for given binding sites compared to Ca^2+^ (Peng et al., 2004; Dudev et al., 2005; Kotelnikova et al., 2021).

First, the La^3+^ initially present in the solution could have facilitated an exchange with the Ca^2+^ present in the soil, resulting in the release of Ca^2+^ into the solution while La^3+^ integrated into the soil. Most of the available Ca^2+^ from the soil might have been replaced this way. Our data supports this idea, because we observed an increasing concentration of available Ca^2+^ in the supernatant of soil slurries when higher amounts of La^3+^ were added, particular in alkaline soil slurry in week 1 (Supplementary Figure S5); and most of the La^3+^ was found in the soil particles of the soil slurries (Figure 4) and less La^3+^ in the supernatant (Supplementary Figure S4).

Second, Ca^2+^ ions in biological cells could have been replaced by La^3+^ ions which has been shown to inhibit cellular functions of microorganisms (Aussel et al., 1996; Mu et al., 2006; Jiang et al., 2008). In addition, the excessive amount of La^3+^, may have blocked Ca^2+^ channels on the cell membrane and subsequently interacting with membrane related enzymes and impairing the functionality of Ca^2+^-dependent processes (Aussel et al., 1996; Wang et al., 2007). These findings suggest that the described processes involving La^3+^ and Ca^2+^ exchange may similarly apply to methanotrophs, potentially influencing their cellular functions and disrupting Ca^2+^-dependent processes.

The low pH in soil slurries from the acidic and neutral rice fields is another possible driving factor of the observed activity and community composition of CH_4_ consuming communities in our laboratory microcosm system. The soil microbiome mainly consuming CH_4_ faced challenges in adapting to acidic conditions (pH = 5.76), limiting the activity of their main MMO through changes in available metal ions such as copper and iron (Yao et al., 2023). However, low pH conditions did not correlate with the observed CH_4_ consumption patterns and are likely not the main explanation for the observed inhibition patterns. For instance, we observed a pH of 4.57 in week 3, but in week 4, the pH in the L treatments in the supernatant of soil slurries from acidic rice fields was 5.86 (see Table 2). However, the CH_4_ consumption activity and community composition remained similar. Therefore, we suggest that the effect of La^3+^ perhaps had a greater impact on CH_4_ consumption at lower pH levels, possibly due to alterations in the solubility and bioavailability of La^3+^.

Next, to pH, other soil properties are important contributing factors that can influence the effect of La^3+^ on the soil microbiome. The mineral composition of the parent material can affect soil cation exchange capacity, which determines the soil’s ability to retain and release nutrients, including La^3+^. In addition, organic matter content plays a crucial role in buffering pH and retaining nutrients. More importantly, organic matter can also bind to La^3+^ ions, affecting their availability to soil microbes (Luo et al., 2021). In our three rice field soils the organic matter content differed substantially, with neutral samples (26.98 g/kg) having the highest concentration, followed by acidic samples (19.48 g/kg), and the lowest in alkaline samples (11.16 g/kg) (Table 1). Soil origin can also influence the availability of essential nutrients, such as nitrogen, potassium, and phosphorus. It is well known that the addition of small quantities of LaCl_3_ forms small precipitates of LaPO_4_ in the presence of phosphates (Ding et al., 2007; Phuruangrat et al., 2012; Silva et al., 2019). As such, higher available phosphorus content in alkaline (37.47 mg/kg) and neutral (33.13 mg/kg) rice field samples may have contributed to the precipitation of La^3+^ thereby changing the bioavailability of La^3+^.

It should be noted that CH_4_ was the sole carbon source added to the soil slurries over the course of the experiment. Our data demonstrated that the soil microbiome simplified in their community composition, with the *Methylobacter* genus as the dominant methanotroph but also non-methanotrophic methylotrophs such as the genus *Methylotenera* and *Methylovorus*. As such, our findings align with previous metagenomic studies that have examined the distribution of carbon derived from CH_4_ across various bacterial populations (Kalyuzhnaya et al., 2008; Hernandez et al., 2015; Oshkin et al., 2015). As shown by Krause et al. (2017) the *Methylobacter* genus can provide carbon in the form methanol to non-methanotrophic methylotrophs such as the *Methylotenera* genus in the presence of La^3+^. Hence, our results further provide evidence for the concept that these communities collectively contribute to CH_4_ cycling, rather than a single type of microorganism. In addition, both methylotrophs are known to possess lanthanide (Ln)-dependent alcohol dehydrogenases (ADH) which may confer an advantage in the presence of high La^3+^ concentrations (Krause et al., 2017; Huang et al., 2018). In this context, we observed that the *Methylocystis* genus, a type II methanotroph, depicted higher relative abundance than the *Methylobacter* genus (Type I) in soil slurries with acidic and neutral rice field soil at higher La^3+^ concentrations (Figure 2). Previous studies already indicated that *Methylocystis* is more flexible than *Methylobacter*, mainly exhibited by their substrate utilization, oxygen requirements, and environmental adaptability (Krause et al., 2015; Hakobyan and Liesack, 2020; Nguyen et al., 2021). Interestingly, the *Methylocystis* genus has been demonstrated to harbor Ln-dependent ADHs (Jung et al., 2020). Our results indicate that *Methylocystis* genus also exhibits increased flexibility under high La^3+^ conditions as well. Hence, we suggest that the soil microbiome involved in CH_4_ consumption can generally cope with the addition of excessive amount of La^3+^ and changes in community composition ensured ecosystem functionality.

While our primary hypothesis lacked support from the data, we did make several noteworthy observations. Firstly, in the fourth week of the experiment, the CH_4_ consumption data revealed a faster decrease in CH_4_ concentration in the L and M treatments when compared to the control treatment for all soil slurries with different initial pH levels (Figure 1). We believe that the experimental design, utilizing only a 1% CH_4_ concentration in the headspace, may have constrained the CH_4_ oxidation potential of these communities and hindered the identification of clear stimulatory effects.

Secondly, we observed an increase in the relative abundance of the genus *Methylobacter* in response to varying levels of added La^3+^ in soil slurries from alkaline rice fields (Figure 2). We conducted qPCR analysis of the *pmoA* gene using the A189F and mb661R primer set (Holmes et al., 1995; Costello and Lidstrom, 1999); however, we were unable to confirm this trend with this primer set. A possible explanation lies in the coverage of the used primer set that fails to detect phylogenetic clusters that fall between *pmoA* and *amoA* (subunit of ammonium monooxygenase) sequences like *pxmA* cluster and high-affinity CH_4_ oxidizers cluster. Previously, the genera *Methylomonas*, *Methylobacter* and *Methylomicrobium* have been shown to a encode a sequence-divergent particulate monooxygenase (pXMO) (Tavormina et al., 2011). In addition, members of the family *Beijerinckiaceae* are not well amplified (Bourne et al., 2001; Knief, 2015). Hence, considering our *pmoA* gene copy number data using A189F/mb661R, it appears that a single primer might not be enough to fully investigate the abundance of methanotrophs in these rice field samples during our experiments, which might not cover all the active methanotrophs.

More importantly, we also did not pick methanotrophs that only have soluble form of the methane monooxygenase (SMMO) next to the pMMO such as *Methyloceanibacter*, which was an essential member of the total microbial community in all our initial soil slurries. Although it is not categorized as a methanotroph, this microbe has been identified to possess a SMMO and gene copies of Ln-dependent PQQ ADHs in marine environments, making it a putative methanotroph that can benefit from the addition of La^3+^ (Vekeman et al., 2016).

It is worth noting another contributing factor to our findings. As mentioned before, the presence of La^3+^ can enhance the activity of methanotrophs by increasing the production of MDH, particularly XoxF, and higher conversion of CH_3_OH into HCHO (Chu and Lidstrom, 2016). However, the metabolites produced in this process may have an impact on the activity of formaldehyde dehydrogenase (FDH) and other enzymes, inhibiting the conversion of CH_4_ into biomass. Consequently, certain intermediates in the CH_4_ oxidation pathway could potentially increase the cell toxicity of the methanotrophs and inhibit overall cell growth.

While our study contributes valuable insights to the ecological effect of added La^3+^ on methanotrophic communities in a microcosm model system, it is essential to acknowledge certain limitations that may impact the interpretation of our findings. One notable constraint is the detection of only approximately 37% of La^3+^ retained in the soil (Supplementary Figure S6). Together with the presence of negligible amounts in the supernatant (approximately 1%), it raises the question about potential precipitation and sequestration mechanisms for the remaining unaccounted fraction (Supplementary Figure S6). As mentioned before La^3+^ has a strong tendency to bind phosphates. Interestingly, we did not detect any PO_4_^3−^ in the supernatant of samples during the experiment (Supplementary Figure S5) supporting this as a mechanism for La^3+^ precipitation. In addition, the portion that remains unaccounted for could also be ascribed to potential interactions within microbial cells, possibly through bioaccumulation. For instance, at an exposure concentration of 20 μM, the La^3+^ concentration within bacteria was measured at a level of 10832 μg g^−1^ dry weight (Zhang et al., 2010). These limitations highlight a need for further studies into the fate and behavior of La^3+^ in soil ecosystems. Rather than relying solely on La^3+^ in the supernatant as a proxy for its bioavailability, employing sequential extractions followed by ICP-MS could enhance our understanding of the unaccounted portion of La^3+^.

## 5 Conclusion

This study offers valuable insights into the potential impacts of increasing amounts of REEs in the form La^3+^ on the soil microbiome originating from rice fields. Our findings imply that the consumption of CH_4_ in soil is influenced by intricate relationships between the soil origin, environmental factors like pH, and the community of microorganisms inhabiting the soil. As such, these findings contribute to a better understanding of the crucial role that microbial communities play in mitigating greenhouse gas emissions and combating climate change. In future research, it is crucial to better account for potential precipitation and bioaccumulation effects and concentrate on the cellular responses of the CH_4_-consuming community. This involves obtaining metagenome-assembled genomes coupled with transcriptomics to elucidate the biological and chemical components during the observed interactions with La^3+^.

## Data availability statement

The data presented in the study are deposited in the National Center for Biotechnology Information (NCBI) repository, BioProject accession number PRJNA1014707.

## Author contributions

RL: Conceptualization, Data curation, Formal analysis, Investigation, Methodology, Validation, Visualization, Writing – original draft, Writing – review & editing. ZW: Writing – original draft, Writing – review & editing. WD: Methodology, Formal analysis, Writing – review & editing. RW: Writing – original draft, Writing – review & editing. JA: Resources, Writing – review & editing. LY: Resources, Writing – review & editing. SK: Conceptualization, Formal analysis, Funding acquisition, Investigation, Methodology, Project administration, Resources, Supervision, Validation, Visualization, Writing – original draft, Writing – review & editing.
