# Supplementary material for: Unraveling the impact of lanthanum on methane consuming microbial communities in rice field soils
Source: Front Microbiol. 2024 Jan 23;15:1298154. doi: 10.3389/fmicb.2024.1298154 (PMC10844099; doi:10.3389/fmicb.2024.1298154)
Supplement: Supplementary file 1 [file Data_Sheet_1.docx]

Supplementary Figures to manuscript:

Unraveling the impact of lanthanum on methane consuming microbial communities in rice field soils

**Ruyan Liu^1^, Ziting Wei^1^, Wanying Dong^1^, Rui Wang^1^, Jonathan M. Adams^2^, Lin Yang^2^, Sascha M.B. Krause^1*^**

^1^School of Ecology and Environmental Sciences, East China Normal University, Shanghai, China

^2^School of Geographic and Oceanographic Sciences, Nanjing University, Nanjing, China

**Supplementary Figure S1.** Top 30 microbes for start material; control (CK), 300 (L), 600 (M), and 1200 (H) mg/L La treatments at the ASV level in three different pH samples, from top to bottom respectively are acidic (a), neutral (b), alkaline (c) samples.

**Supplementary Figure S2.** β-diversity for start material, control (CK), 300 (L), 600 (M), and 1200 (H) mg/L La treatments in acidic (a), neutral (b), and alkaline (c) samples over time.

**Supplementary Figure S3.** Shannon diversity in three samples at different pH for start material, control (CK), 300 (L), 600 (M), and 1200 (H) mg/L La treatments (*, *P* < 0.05; **, *P* < 0.01; ***, *P* < 0.001; ****, *P* < 0.0001; ns, no significance). Distinct letters in the plot indicate statistically differences among different La^3+^ treatments (*P*<0.05).

**Supplementary Figure S4.** The concentration of La in the supernatant for control (CK), 300 (L), 600 (M), and 1200 (H) mg/L La treatments (*, *P* < 0.05; **, *P* < 0.01; ***, *P* < 0.001; ****, *P* < 0.0001; ns, no significance). Error bars indicate SD (n=3), distinct letters in the plot indicate statistically differences among different La^3+^ treatments (*P*<0.05).

**Supplementary Figure S5.** Contents of NH_4_^+^, Ca^2+^, NO_3_^-^, and PO_4_^3-^ in the supernatant for control (CK), 300 (L), 600 (M), and 1200 (H) mg/L La treatments (*, *P* < 0.05; **, *P* < 0.01; ***, *P* < 0.001; ****, *P* < 0.0001; ns, no significance). Error bars indicate SD (n=3), distinct letters in the plot indicate statistically differences among different La^3+^ treatments (*P*<0.05).

**Supplementary Figure S6.** Fractions of La^3+^ content recovered after 4 weeks of incubation in soil particles and supernatant of 300 (L), 600 (M), and 1200 (H) La treatments.


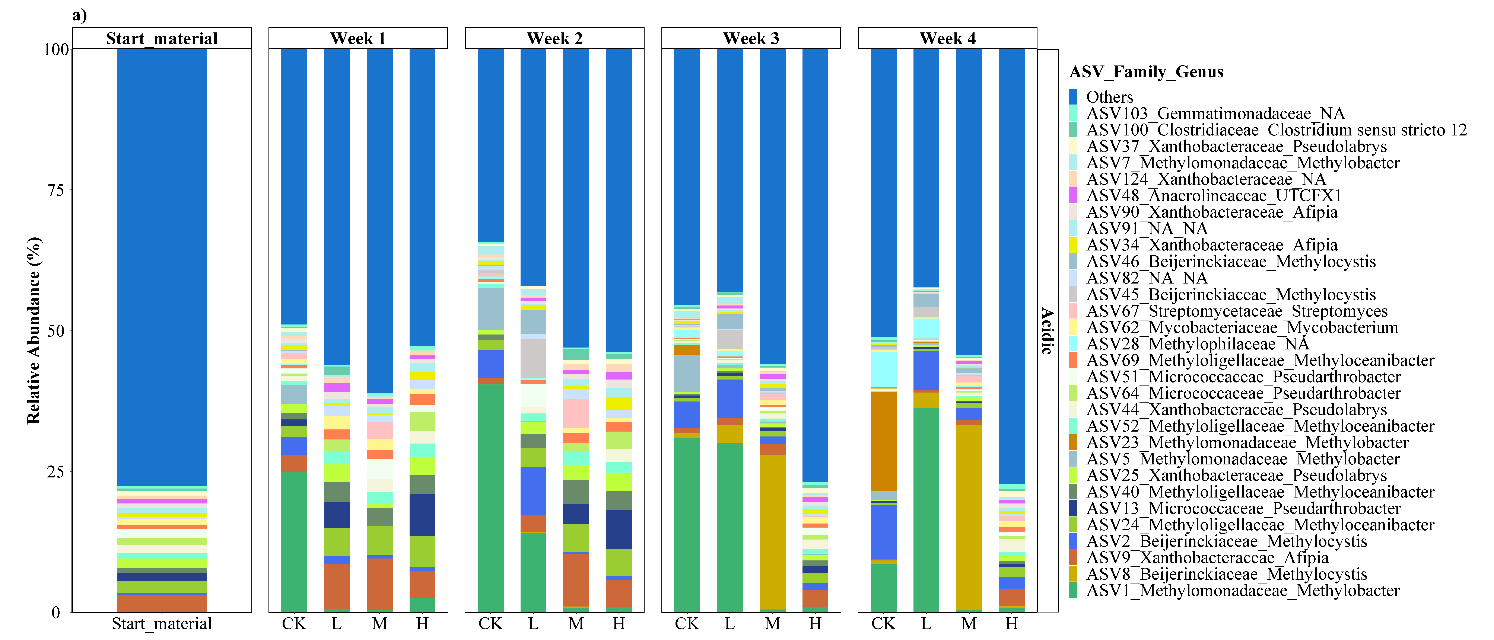


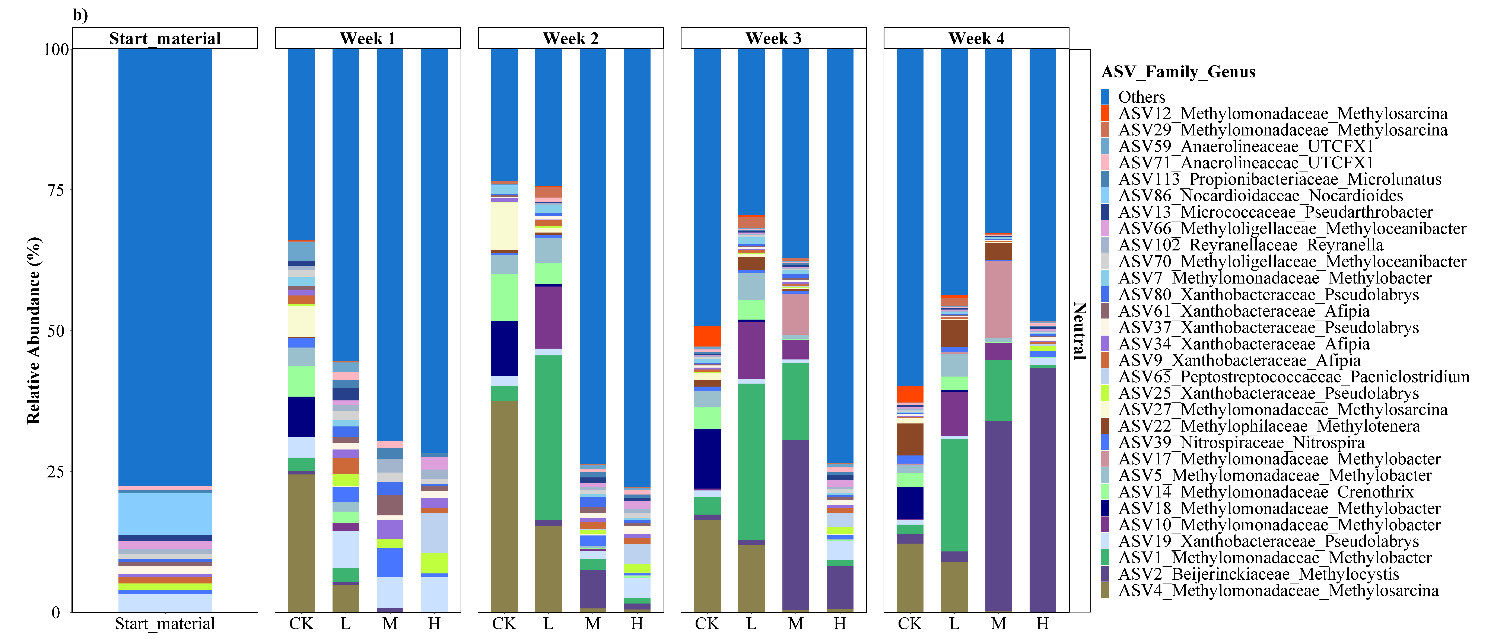

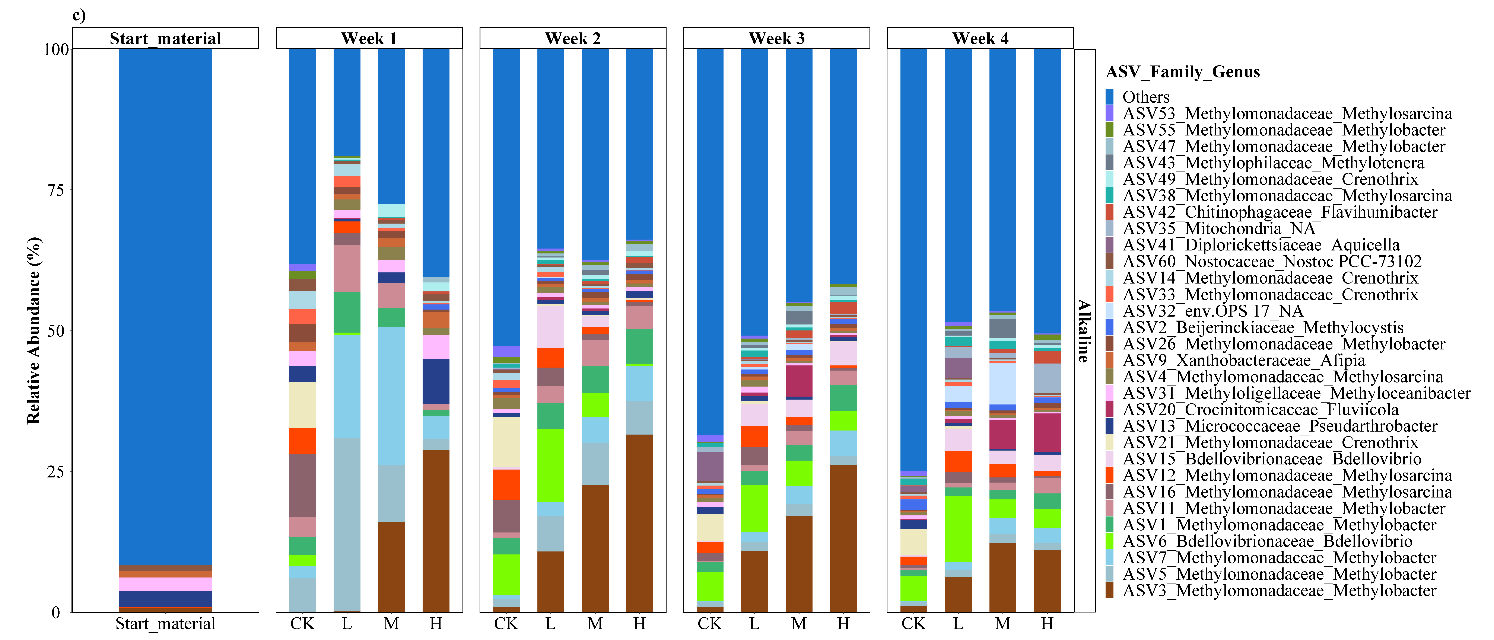


**Supplementary Figure S1.** Top 30 microbes for start material; control (CK), 300 (L), 600 (M), and 1200 (H) mg/L La treatments at the ASV level in three different pH samples, from top to bottom respectively are acidic (a), neutral (b), alkaline (c) samples.


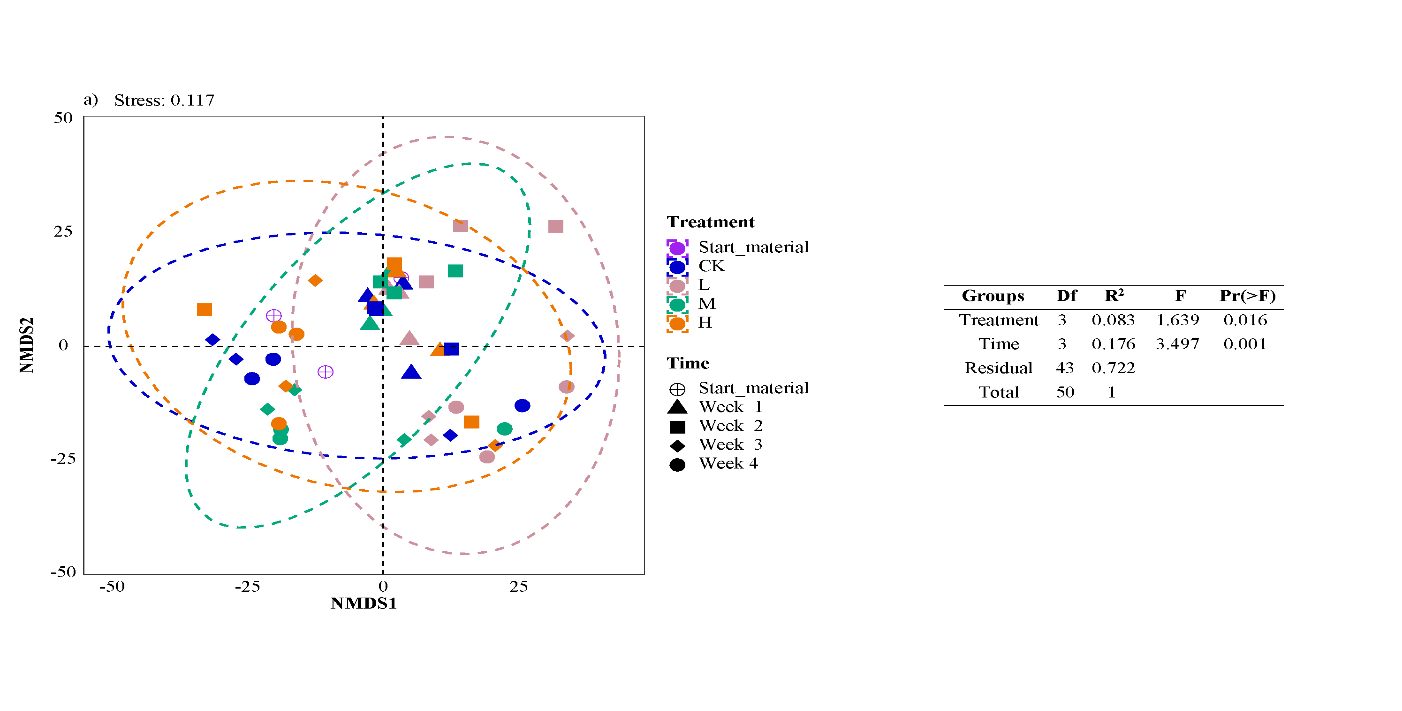


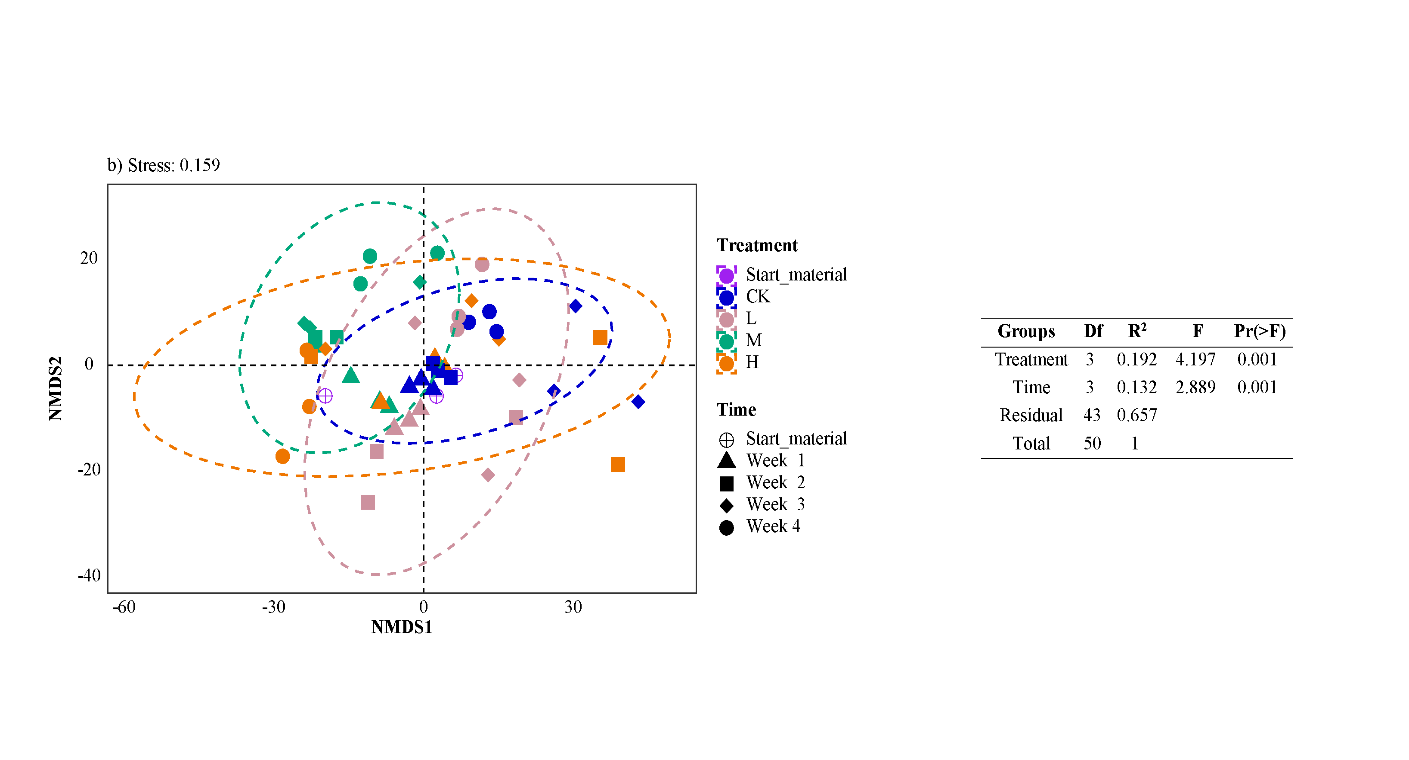


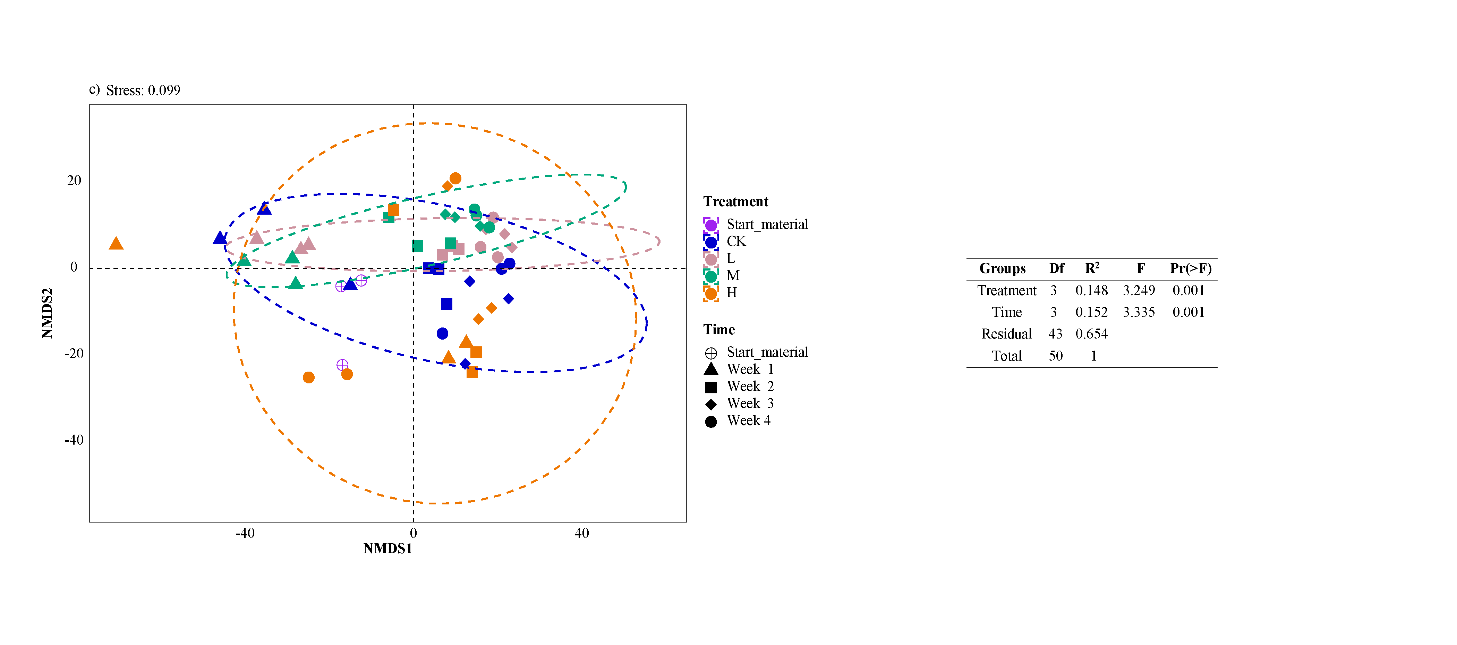
**Supplementary Figure S2.** β-diversity for start material, control (CK), 300 (L), 600 (M), and 1200 (H) mg/L La treatments in acidic (a), neutral (b), and alkaline (c) samples over time.


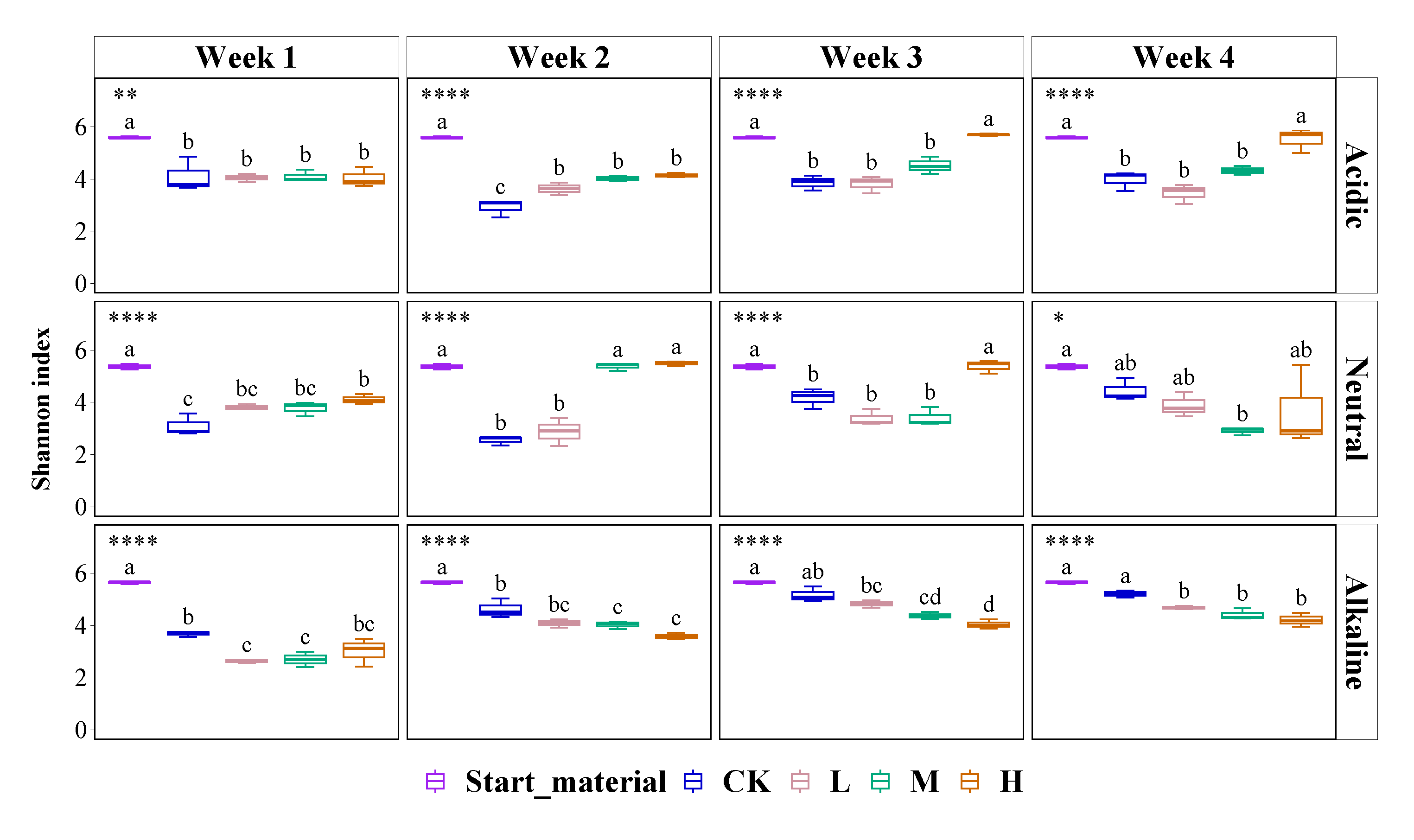


**Supplementary Figure S3.** Shannon diversity in three samples at different pH for start material, control (CK), 300 (L), 600 (M), and 1200 (H) mg/L La treatments (*, *P* < 0.05; **, *P* < 0.01; ***, *P* < 0.001; ****, *P* < 0.0001; ns, no significance). Distinct letters in the plot indicate statistically differences among different La^3+^ treatments (*P*<0.05).


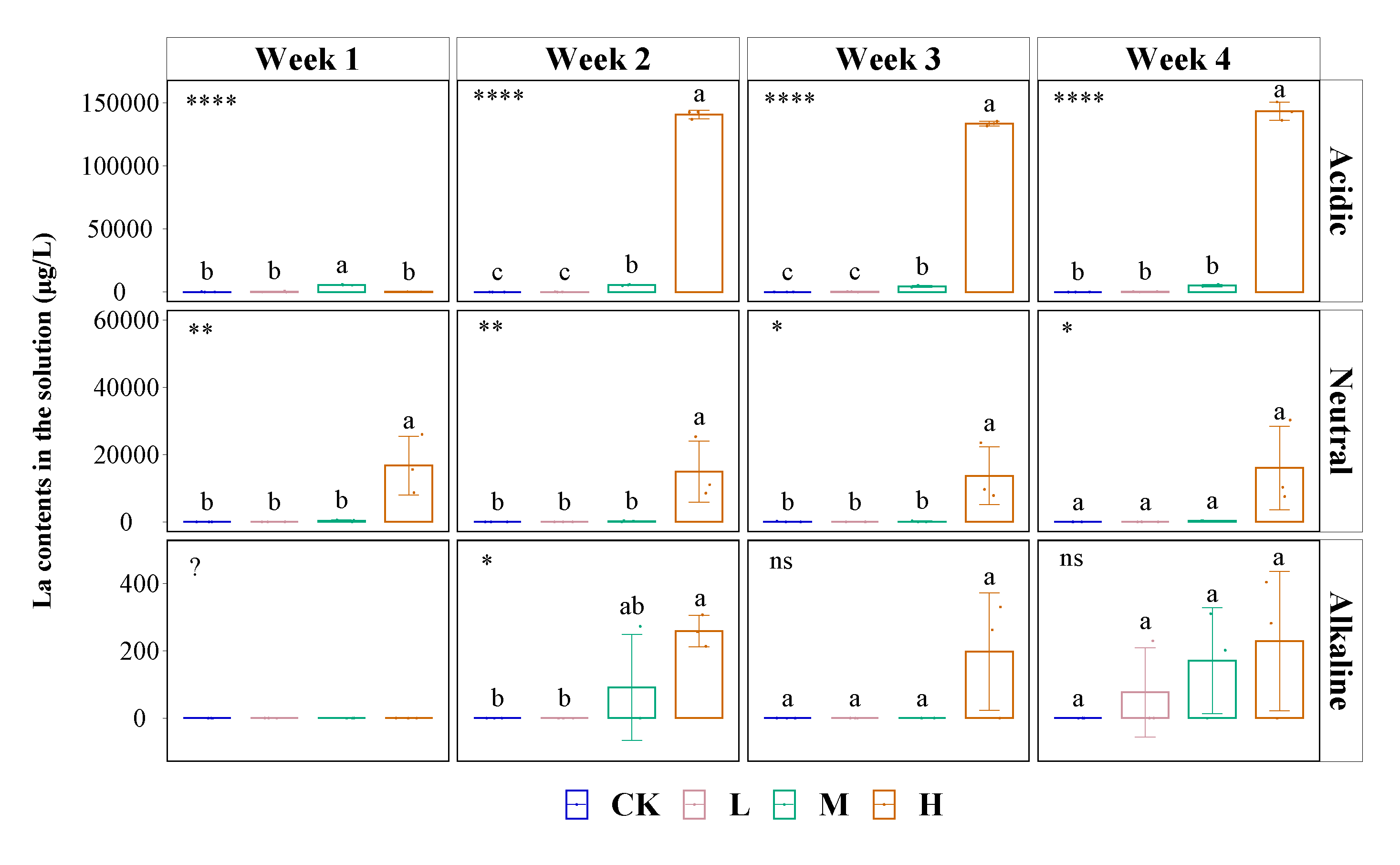


**Supplementary Figure S4.** The concentration of La in the supernatant for control (CK), 300 (L), 600 (M), and 1200 (H) mg/L La treatments (*, *P* < 0.05; **, *P* < 0.01; ***, *P* < 0.001; ****, *P* < 0.0001; ns, no significance). Error bars indicate SD (n=3), distinct letters in the plot indicate statistically differences among different La^3+^ treatments (*P*<0.05).


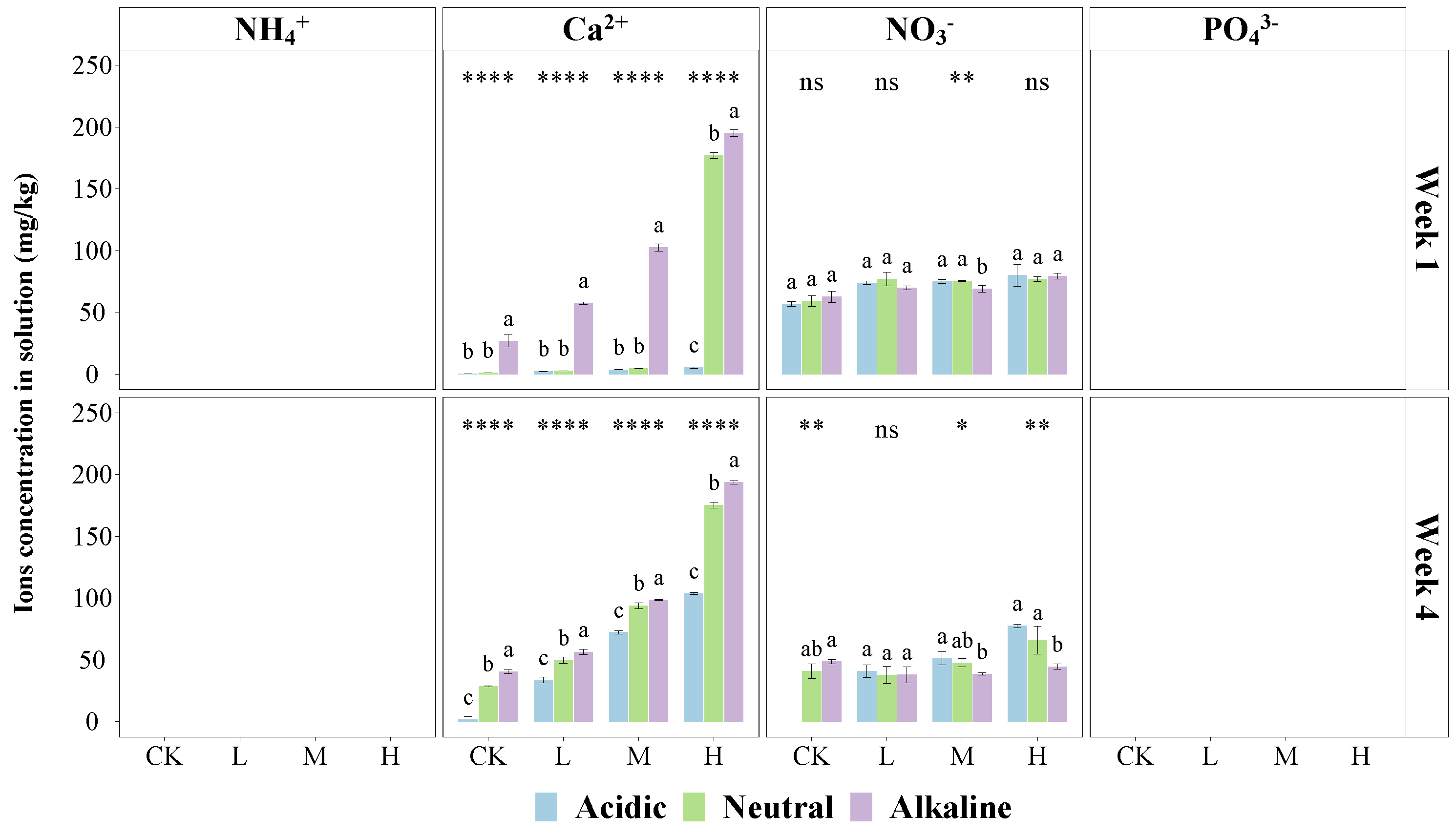


**Supplementary Figure S5.** Contents of NH_4_^+^, Ca^2+^, NO_3_^-^, and PO_4_^3-^ in the supernatant for control (CK), 300 (L), 600 (M), and 1200 (H) mg/L La treatments (*, *P* < 0.05; **, *P* < 0.01; ***, *P* < 0.001; ****, *P* < 0.0001; ns, no significance). Error bars indicate SD (n=3), distinct letters in the plot indicate statistically differences among different La^3+^ treatments (*P*<0.05).


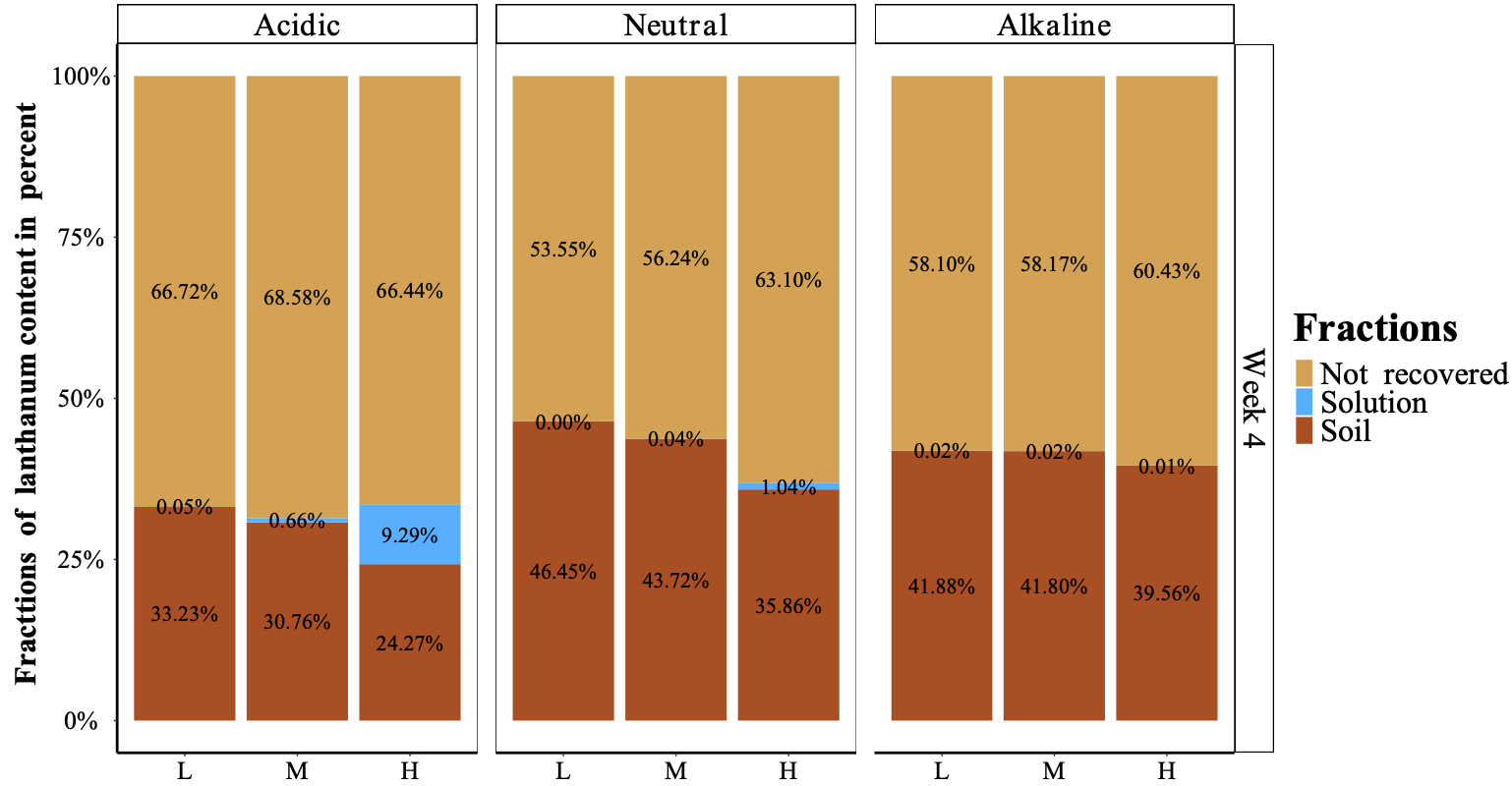


**Supplementary Figure S6.** Fractions of La^3+^ content recovered after 4 weeks of incubation in soil particles and supernatant of 300 (L), 600 (M), and 1200 (H) La treatments.
